# Proficient One-Step Heat-Up Synthesis of Manganese Sulfide Quantum Dots for Solar Cell Applications

**DOI:** 10.3390/molecules27196678

**Published:** 2022-10-07

**Authors:** Mojeed A. Agoro, Edson L. Meyer

**Affiliations:** 1 Fort Hare Institute of Technology, Faculty of Science and Agriculture, University of Fort Hare, Private Bag X1314, Alice 5700, Eastern Cape, South Africa; 2 Department of Chemistry, University of Fort Hare, Private Bag X1314, Alice 5700, Eastern Cape, South Africa

**Keywords:** energy device, manganese sulfide, heat-up route, morphology, size distribution

## Abstract

The necessity to develop renewable energy resources that are highly durable and flexible with superior energy density and capacitance ability has attracted considerable interest in the field of solar cell research. Semiconducting compound materials that are easily available, hazard-free and cost-effective are emerging as potential solutions to tackle this challenge. Herein, we present multiple molecular precursors used to grow manganese sulfide nanoparticles through a proficient one-step heat-up approach. For all of the tested samples, the X-ray diffraction peaks correspond to a γ-MnS hexagonal wurtzite structure. UV-Vis spectroscopy yielded absorption wavelengths of 359–420 nm and band-gap energies of 3.78–4.0 eV. Photoluminescence analysis shows characteristics of red and blue shift from 451–602 nm. High-resolution transmission electron microscopy (HRTEM) and selected-area electron diffraction (SAED) reveal a narrow size distribution with nanosticks and large contact areas, which are critical for improved catalytic performance. The current study provides an improved pathway to a well-grown and uniform nanocrystal structure for applications in energy devices.

## 1. Introduction

Semiconductor quantum dots (QDs) employed as photosensitizers with tunable band gaps as an alternative to dye-sensitized solar cells (DSCs) has attracted increasing attention from researchers in recent years. QDs have a unique absorption coefficient for large light, with high stability due to their tunable band gap [1,2,3,4,5]. The use of QD photosensitizers from inorganic semiconductors is gaining increasing attention, with more studies being conducted due to their advantages relative to homologue dyes [1,2,3]. Owing to their physical and chemical properties, chalcogenide metal compounds, such as manganese, have recently received a considerable amount of attention as modern, innovative technology in a variety of fields [6]. The growth conditions of QD semiconductors can be altered via techniques to attain desirable optical, structural, electrical and morphological properties [7,8]. Magnetic semiconductor manganese sulfide (MnS) has a band gap of (Eg = 3.1–3.7 eV) and considerable potential in applications such as blue–green light emitters, optoelectronic devices, solar cells, photoconductors, sensors, coatings and mass optical memories [9,10,11,12]. They grow at lower temperatures and come in three structural shapes: zinc-blende, rock-salt and wurtzite. The rock-salt MnS structure is more stable than both β and γ-MnS and can irreversibly transform into a stable form of rock-salt structure at high temperatures of 100–400 °C [13,14,15,16], accounting for the unique chemical properties displayed by metastable MnS as compared to the stable phase [8].

Various approaches have been reported for the preparation of MnS through hydrothermal, solvothermal, chemical bath deposition (CBD), chemical vapor deposition (CVD) and successive ionic layer adsorption and reaction (SILAR) [17,18]. A single-source method produces high-purity and cost-effective materials by incorporating the required elements into one compound [1,2,3]. This approach involves a simple decomposition of the molecular precursor, usually dithiocarbamate metal complexes, in an inert environment to the desired temperature [19,20,21]. Such approaches have been employed for the formation of metal oxides and metal chalcogenides with various morphologies for different nanomaterials, such as nanofabrics, nanodiscs, nanorods, nanospheres and nanowires [22,23]. In the present study, we report on the formation of MnS through a single-source precursor approach. We investigated roles of aromatic mixed and single ligands in the MnS structural, optical, morphological and thermal stability.

## 2. Results and Discussion

### 2.1. Thermogravimetric Analysis (TGA) of Precursors **PR1**, **PR2** and **PR3**

**Figure 1:**
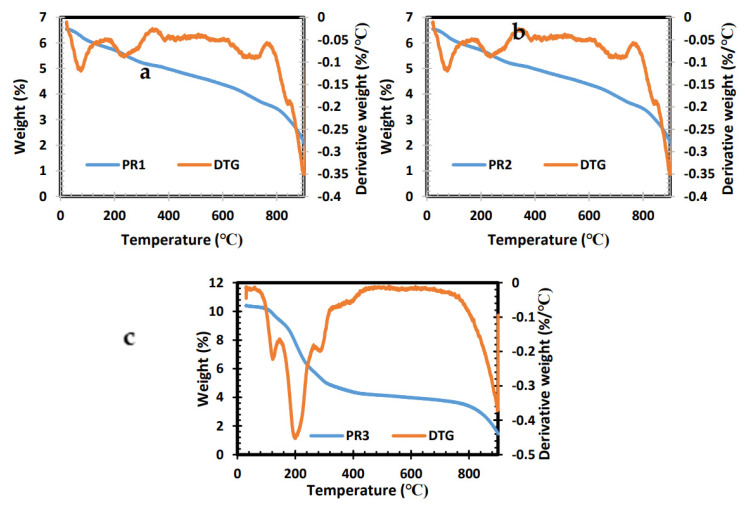
TGA and derivative thermogravimetric (DTG) analysis of **PR1** (**a**), **PR2** (**b**) and **PR3** (**c**) complexes.

Thermogravimetric elevation within the temperature range of 30–900 °C was carried out to determine the thermal properties of the complexes shown in Figure 1b,c. **Mn[*N*-piper-*N*-*p*-anisdtc] (PR1)** and **Mn[*N*-piperdtc] (PR2)** exhibit similar properties in their plots, with mass loss of 5.1% between 321 °C and 330 °C. The second step involved evaluation at a temperature of 746 °C and 740 °C, with a mass loss of 3.7% and 3.6%, signifying the complete removal of the phenyl compound moiety. The TGA plot for **Mn[*N*-*p*-anisdtc] (PR3)** illustrates three steps of degradation at 183 °C, 322 °C and 446 °C, with a final residual of 4.2% as MnS [24].

### 2.2. XRD

**Figure 2:**
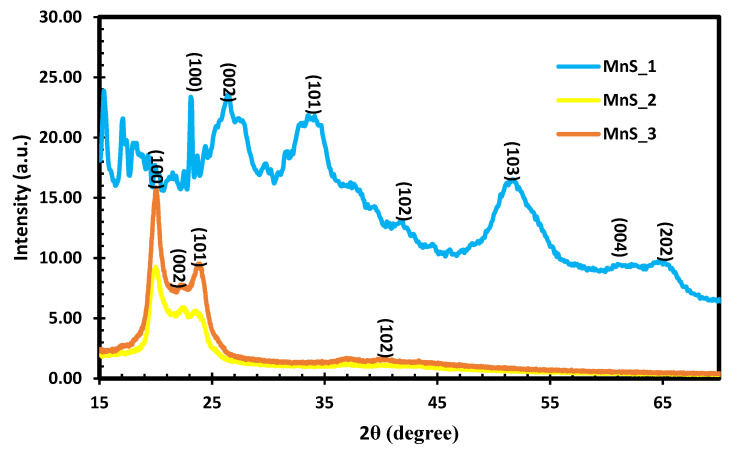
XRD of MnS_1, MnS_2 and MnS_3 nanoparticles.

Dithiocarbamate complexes are suitable precursors that are usually stable at room temperature but decompose under high heat to form metal sulfide. The XRD reveals the structural pattern of the prepared MnS nanoparticles using three molecular precursors. The diffraction peaks obtained from the XRD analysis are illustrated in Figure 2 for MnS_1, MnS_2 and MnS_3. The obtained peaks are consistent with the γ-MnS hexagonal wurtzite structure data standards (JCPDS Card No. 40–1289). The XRD results obtained in this study are in agreement with previous reports [25,26,27] on the γ-MnS wurtzite phase. The XRD patterns of the three samples at 260 °C show disperity in intensity that can be attributed to differences in carbon ring molar concentration in the molecular precursor with manganese ions [28,29]. Furthermore, the γ-MnS phase, nanocrystalline reductions and structural distortion were previously reported as a result of the Jahn–Teller effect at room temperature, as well as the oxygen content [28,29]. 

### 2.3. FTIR

**Figure 3:**
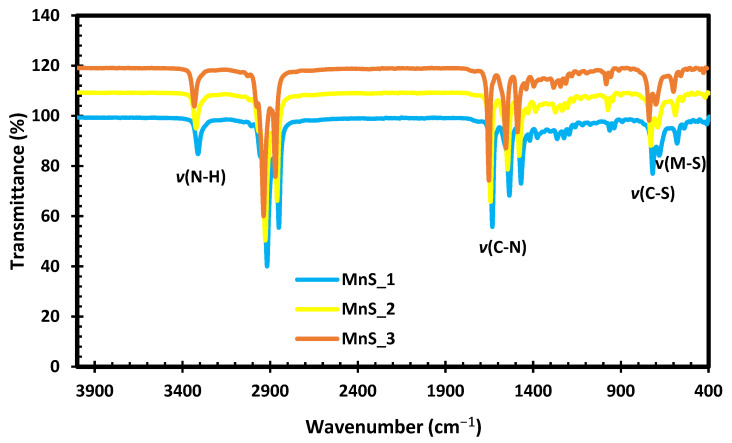
FTIR spectra of MnS_1, MnS_2 and MnS_3 nanoparticles.

As shown in Figure 3, the three samples of MnS have the same intensities of vibrations at various peaks. The vibration peaks at 580 cm^−1^ are related to Mn-S vibration modes in all the samples, as similarly reported in [30]. The low-intensity bands observed around 400–500 cm^−1^ are attributed to the interaction between resonances of the sulfide ions. C-S linkage vibration modes are observed at 720 cm^−1^ for all the samples, whereas C-N peaks occur at 1471–1634 cm^−1^ [31]. The high-intensity bands observed around 2918–2852 cm^−1^ are ascribed to the sp^1^ (C-H) group in phenyl units [30], whereas the peaks at 3314 cm^−1^ correlate to the N-H stretching modes for the three MnS samples.

### 2.4. UV-Vis Spectroscopy

**Figure 4:**
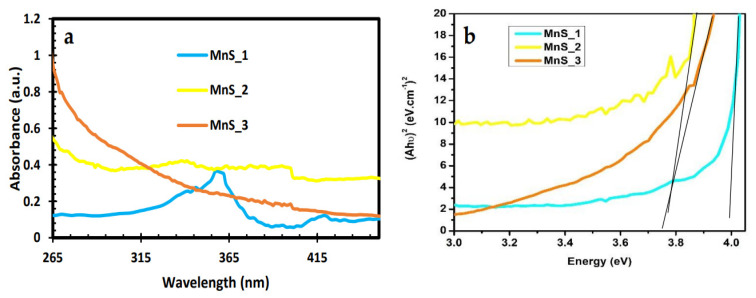
UV-Vis spectra (**a**) and Tauc plot with extrapolated linear line (**b**) of MnS_1, MnS_2 and MnS_3 nanoparticles.

The optical behavior of the fabricated MnS_1, MnS_2 and MnS_3 nanoparticles was evaluated via UV–Vis spectroscopy. Both MnS_2 and MnS_3 exhibit absorption properties at 402 nm with a wavelength in the visible regions, as indicated in Figure 4a,b, with a band-gap energy of 3.78 eV. Unlike the other two samples, MnS_1 displays a slit edge with an absorption response at 359 nm and 420 nm and an energy band gap at 4.0 eV. This slit edge can be linked to the carbon ring molar concentration in the molecular precursor with manganese ions, which is a similar trend as that observed in the XRD results.

### 2.5. PL

**Figure 5:**
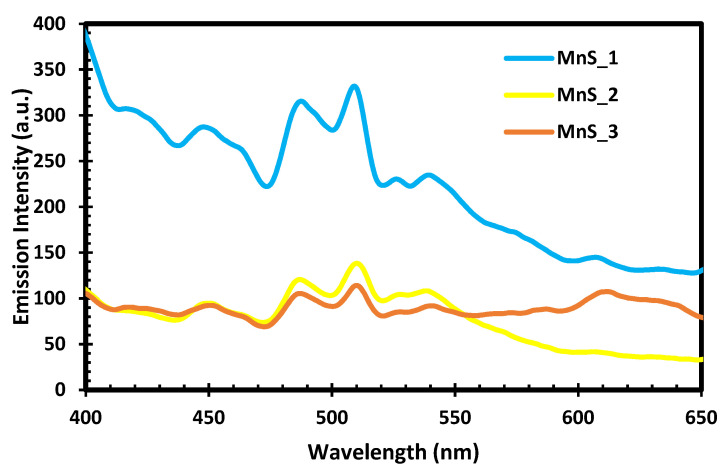
PL spectra of MnS_1, MnS_2 and MnS_3 nanoparticles.

The photoluminescence spectra of MnS_1, MnS_2 and MnS_3 are shown in Figure 5. All the samples have the same emission peaks at 451 nm, 490 nm, 511 nm, 527 nm, 541 nm and 609 nm at 365 nm excitation. The observed emission peaks at 451 nm and 490 nm were assigned to the blue shift and band-edge emission involving the excited states of the Mn^2+^ ions [27]. The emission peak at 511 nm was attributed to the green shift of manganese and sulphur vacancies associated with interstitial defects [32,33]. Three other peaks at 527 nm, 541 nm and 609 nm are ascribed to the octahedral-coordinated Mn^2+^ ion excitation, which in agreement with the results reported in [32,33]. The emission peaks can be attributed to the radiative recombination of electrons in surface-state shallow traps with photogenerated holes caused by lattice-stacking faults, in agreement with the stacking faults identified by HRTEM [27,34].

### 2.6. HRTEM and SAED

**Figure 6:**
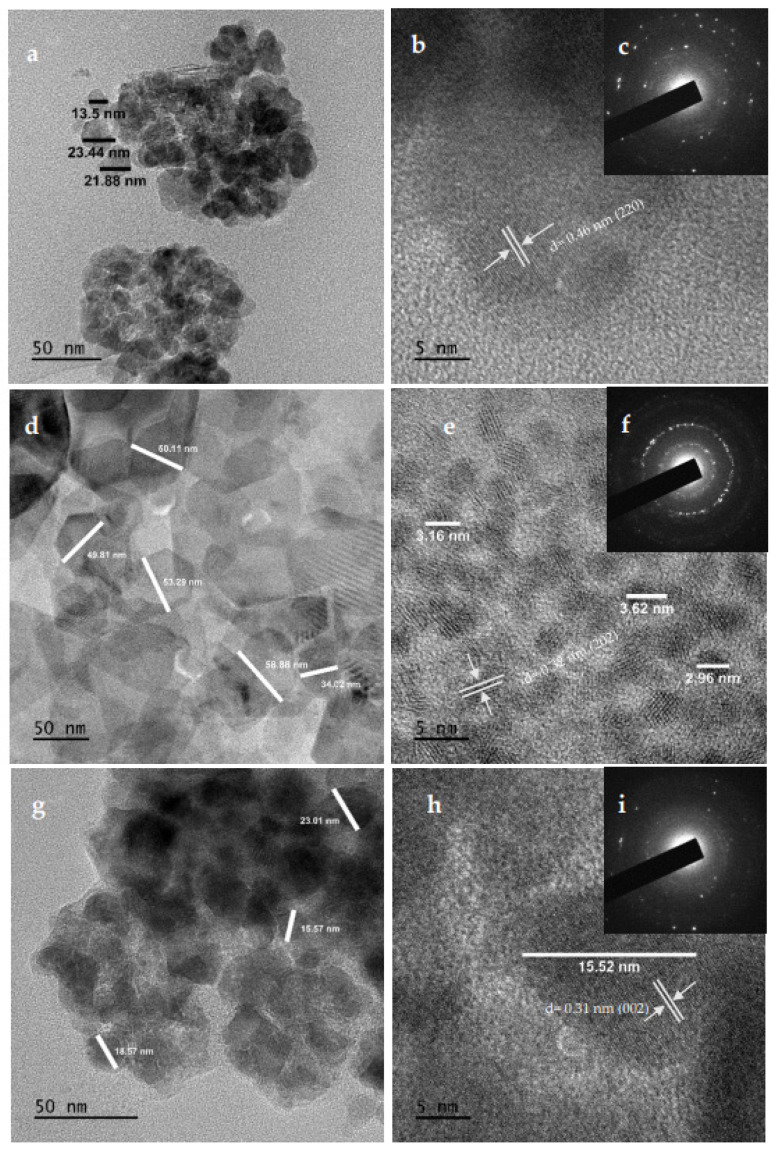
HRTEM and SAED images of MnS_1 (**a,b**) and (**c**), MnS_2 (**d**,**e**) and (**f**) and MnS_3 (**g**,**h**) and (**i**) nanoparticles.

HRTEM and selected area electron diffraction (SAED), as shown in Figure 6c,f,i were used to identify the crystalline structure and size distribution of well-grown MnS_1, MnS_2 and MnS_3 nanoparticles. MnS_1 and MnS_3 exhibit a narrow size distribution between 13.5–23.88 nm and 15.52 nm with nanosticks, indicating improved catalytic performance due to the large contact area, as shown in Figure 6a,b,g,h. The inserted SAED shown in Figure 6c shows a lattice d spacing of 0.46 nm, which is indexed to the MnS crystal plane of 220. MnS_3 shows an interplanar distance of 0.32 nm as obtained from the lattice fringes (Figure 6i), corresponding to the 002 planes of MnS. This is also affirmed by the inserted image in Figure 6i of the SAED pattern. The inner hollow structure, as well as the outer and inner surfaces with a smooth and relatively dense surface, are confirmed by Figure 6d,e for MnS_2, with a particle size of 2.96–58.88 nm. The SAED pattern shown in the insert in Figure 6f indicates a high crystallinity. The lattice fringes shown in Figure 6e with a d spacing of 0.31 nm could be indexed to the 002 plane of the MnS crystal. The HR-TEM images are in conformity with the respective XRD planes and supported by the results of previous studies [34,35,36]. A summary of the samples prepared through the single-source precursor route is presented in Table 1.

### 2.7. FESEM and EDS

**Figure 7:**
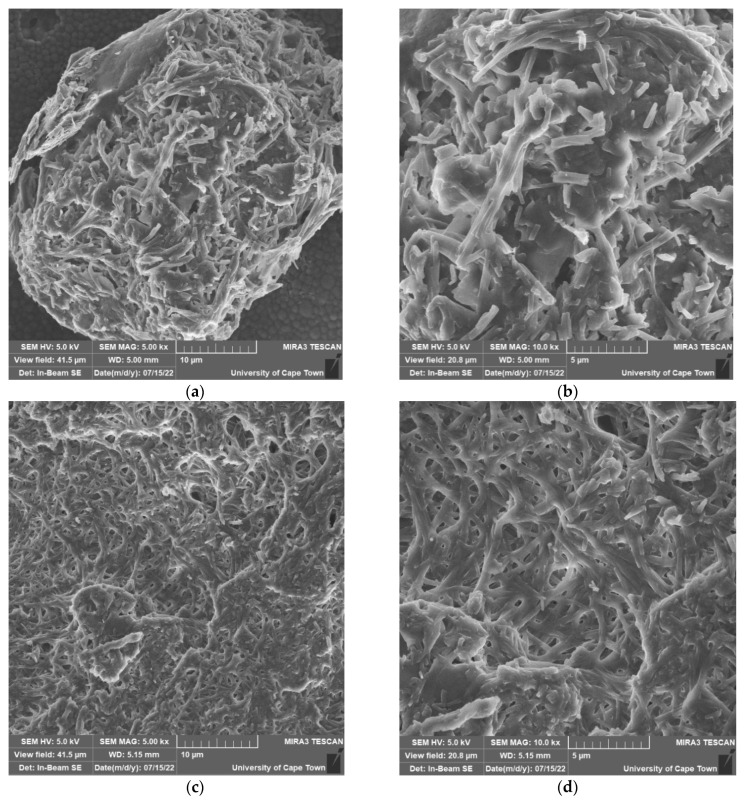

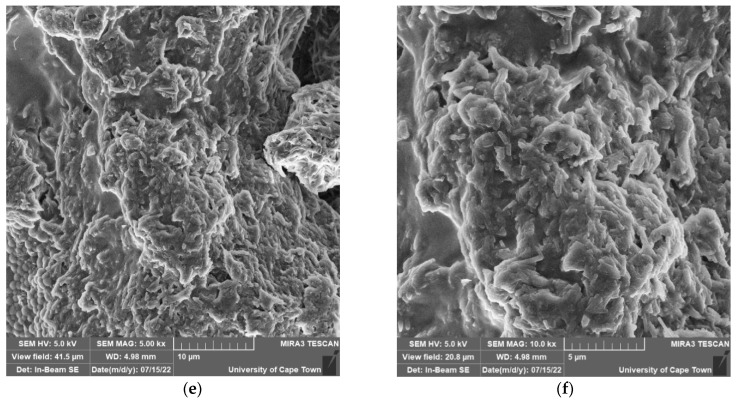
FESEM images of MnS_1 (**a**,**b**), MnS_2 (**c**,**d**) and MnS_3 (**e**,**f**) nanoparticles.

**Figure 8:**
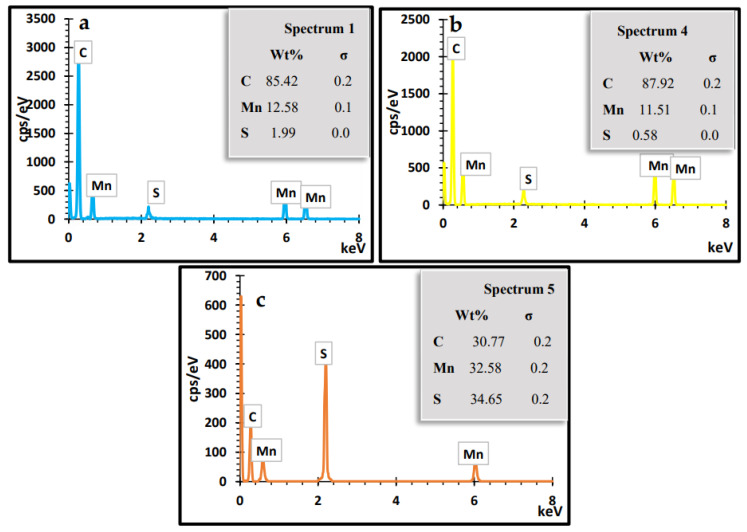
EDS spectra of MnS_1 (**a**), MnS_2 (**b**) and MnS_3 (**c**) nanoparticles.

The microstructure and morphology of MnS_1, MnS_2 and MnS_3 nanoparticles were determined from SEM images and are displayed in Figure 7a–f. The FESEM images for MnS_1 reveal a spongy spherical microstructure with small nanoparticles (see Figure 6a,b) in agreement with the HRTEM results. The FESEM morphology of the MnS_2 and MnS_3 samples consists of spherical shapes and other irregular elongated shapes of numerous particles, as shown in Figure 6c–f. The SEM results followed a similar pattern as the XRD, further affirming the SEM results. [37,38]. The quantitative and qualitative characteristics of the three samples were taken from the EDS spectrum to determine the elemental composition of the MnS nanoparticles, as shown in Figure 8a–c. The EDS clearly reveals the purity of the three MnS nanoparticles, with the presence of elements such as Mn, C and S with no other elements [37].

In this study, we employed a single-source precursor using MnCl₂·4H₂O and TOPO as coordinating solvents in a single step to form γ-MnS nanoparticles, representing a cost-effective method to address the stoichiometry retention challenge of γ-MnS during the deposition process for enhanced and stable photovoltaic performance. Balpinar and Göde [39] reported an efficiency of 3.40–2.28% for CdS doped with Mn with a band gap of 2.41–2.43 eV. According to Akman’s [40] research, doping the Mn/ZnO photoanode in solar cell applications resulted in stability and an improvement in efficiency of 2.11–4.20% for 3.34-3.19 eV. Fadaam et al. [41] demonstrated enhanced conversion for MnS through annealing at 300 °C with a band-gap energy of 2.9 eV. Cao et al. [42] reported on the heterojunction structure of NiS2/MnS with 6.44 % and 4.81% for MnS FTO as a potential counter electrode to replace Pt as a low-cost and highly efficient material for dye-sensitized solar cells. Based on electrochemical, structural and optical analyses, the γ-MnS phase was investigated as an anode material and as efficient visible light emitters with improved cycling stability for Li-ion batteries [43,44,45]. The band-gap energy at 3.78–4.0 eV and the high emission intensity in the present study are strong indications that these materials can absorb the entire solar spectrum, improving efficiency in solar cell applications. Furthermore, the irregular protrusions on the surface are vital aspects of materials that can enhance electrolyte transport and photoactivity and optimize light absorbance.

## 3. Materials and Methods

### 3.1. Materials

All chemicals were of analytical grade, purchased from Sigma-Aldrich, (Johannesburg, South Africa), and used without further purification, including methanol, carbon disulfides, diethyl ester, ammonium, hexadecylamine (HDA), oleic acid, *p*-anisidine, piperidine and manganese(II) chloride tetrahydrate (MnCl_2_·4H_2_O).

### 3.2. Synthesis of Dithiocarbamate Molecular Precursors

#### Synthesis of Ammonium *N*-Piperidinyldithiocarbamate

Ammonium *N*-piperidinyldithiocarbamate was prepared according to the literature [1]. A volume of 9.9 mL (0.1 mol) of piperidine was added to 30 mL of ammonium solution with 6.04 mL (0.1 mol) of carbon disulfide in a dropwise reaction at 5 °C and stirred for 1 h to precipitate. A solid yellow precipitate was formed. The precipitate was filtered and washed with diethyl ester and dried in the air, which resulted in the final product of ammonium *N*-piperidinyldithiocarbamate labelled as **[*N*-piperldtc]**(16.67 g, 96.7%). Mp: 93–94 °C, ^1^H NMR (DMSO) δ 6.47–7.46 (m, 8H-C_6_H_5_), 4.48–5.32 (s, 2H-NH), 2.50 (s, 1H-SH). ^13^C NMR (DMSO) δ40 (-NH_2_), 55.4 (-S-C), 121-157 (-8H-C_6_H_5_), 204 (-CS_2_). Selected IR (cm^−1^) 1412 *v*(C-N), 1219 *v*(C-S), 3419 *v*(N-H). UV–Vis (CH_3_OH solution, nm): 315. The same method was used for the ammonium *N*-anisidinyldithiocarbamate labeled as **[*N*-*p*-anisildtc]** (20.20 g, 97.6%). Mp: 94–98 °C, ^1^H NMR (DMSO) δ 7.32–9.42 (m, 8H-C_6_H_5_), 3.63–3.75 (s, 2H –NH), 6.91 (s, O-C_6_H_5_), 2.51 (s, 1H-SH). ^13^C NMR (DMSO) δ40 (-NH_2_), 55.7 (-S-C), 180.7 (s, O-C_6_H_5_), 126.6–142.5 (-8H-C_6_H_5_), 208 (-CS_2_). Selected IR (cm^−1^) 1412 *v*(C-N), 1218 *v*(C-S), 3391 *v*(N-H). UV–Vis (CH_3_OH solution, nm): 327.

### 3.3. Synthesis of Bis(N-Piperl-N-p-Anisildithiocarbamato)Manganese(II) Complexes, **Mn[N-Piper-N-p-Anisdtc] (PR1)**

**Scheme 1:**
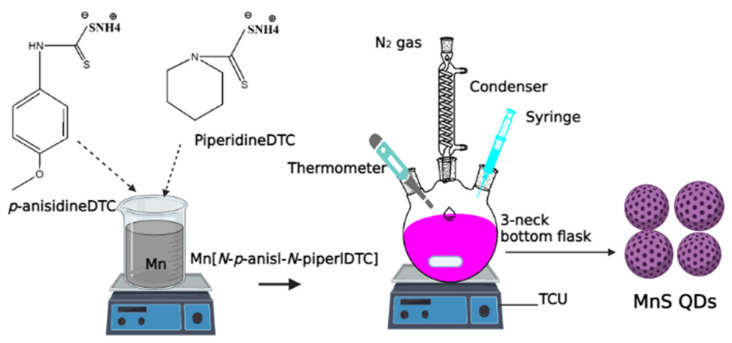
Preparation of MnS_1, MnS_2 and MnS_3 QDs.

MnCl₂·4H₂O salt (0.3147 g, 2.5 mmol) dissolved in (15 mL) was slowly added to (0.4459 g, 2.5 mmol and 0.5409 g, 2.5 mmol) *N*-anildtc and *N*-p-anisildtc ligands dissolved in 15 mL distilled water under stirring in a 1:1:1 ratio. A dark brown precipitate was observed. The mixture was stirred constantly at room temperature for 1 h. The dark brown precipitate was filtered and washed with distilled water three times and dried under a calcium vacuum overnight, as shown in Scheme 1, formulated as **Mn[*N*-anil-*N*-p-anisdtc] (PR1)**. Yield: (6.89 g, 89.2%). Mp: 256–259 °C, ^1^H NMR (DMSO) δ 7.31–9.49 (m, 8H-C_6_H_5_), 3.71 (s, 2H-NH), 6.91 (s, O-C_6_H_5_), 2.50 (s, 1H-SH). ^13^C NMR (DMSO) δ40 (-NH_2_), 55.9 (-S-C), 180.9 (s, O-C_6_H_5_), 114.1–157 (-8H-C_6_H_5_), 208 (-CS_2_). Selected IR (cm^−1^) 1500 *v*(C-N), 1231 *v*(C-S), 3216 *v*(N-H), 515–613 *v*(M-S). UV–Vis (CH_3_OH solution, nm): 308. The same procedure was applied for the synthesis of *N*-piperidinyldithiocarbamate with MnCl₂·4H₂O at ratio of 2:1 formulated as **Mn[*N*-piperdtc] (PR2)** and *N*-*p*-anisidinyldithiocarbamate with MnCl₂·4H₂O at ratio of 2:1, formulated as **Mn[*N*-p-anisdtc] (PR3)**. **(PR2)** Yield: (7.88 g, 84.8%). Mp: 250–253 °C. ^1^H NMR (DMSO) δ 7.31–9.44 (m, 8H-C_6_H_5_), 3.75 (s, 2H–NH), 6.89 (s, O-C_6_H_5_), 2.5 (s, 1H-SH). ^13^C NMR (DMSO) δ40 (-NH_2_), 55.7 (-S-C), 180.7 (s, O-C_6_H_5_), 114.1–157 (-8H-C_6_H_5_), 204 (-CS_2_). Selected IR (cm^−1^) 1500 *v*(C-N), 1232 *v*(C-S), 3165 *v*(N-H), 468–613 *v*(M-S). UV–Vis (CH_3_OH solution, nm): 299. **(PR3)** Yield: (8.22 g, 87.8%). Mp: 250–254 °C. ^1^H NMR (DMSO) δ 7.4–9.4 (m, 8H-C_6_H_5_), 4.22 (s, 2H –NH), 2.50 (s, 1H-SH). ^13^C NMR (DMSO) δ40 (-NH_2_), 51.2 (-S-C), 126.5-157 (-8H-C_6_H_5_), 206 (-CS_2_). Selected IR (cm^−1^) 1500 *v*(C-N), 1232 *v*(C-S), 3166 *v*(N-H), 488-613 *v*(M-S). UV–Vis (CH_3_OH solution, nm): 317 [1]. 

### 3.4. Synthesis of MnS Metals Sulfide Nanoparticles

The MnS metal sulfide nanoparticles were fabricated by a simple one-step, one-pot technique with the aid of three reagents in 2 h. In a typical synthesis under an inert atmosphere, 0.2 g of **PR1** dissolved in 4 mL of oleic acid was mixed with 3 g of hot HDA coordination solvent inserted into a three-neck bottom flask with a magnetic stirrer, cooler reflux and a thermometer. The HDA was heated to between 20 and 30 degrees Celsius before being raised to 260 degrees Celsius. The reaction was maintained for 1 h at the desired temperature. The dark solution was collected by allowing the temperature to drop to 70 °C, then adding about 50 mL of methanol before undergoing centrifugation at 2000 rpm for 30 min to remove the excess coordinating solvent. Oleic acid was then dried in a vacuum. The final product of MnS was formulated as MnS_1. The same procedures were followed for MnS_2 from **PR2** and MnS_3 from **PR3**, as shown in Scheme 1 [1,2,3].

### 3.5. Materials Characterizations

The optical properties, surface morphology, thermal stability, elemental compositions and thickness of the MnS nanoparticles were evaluated through the following techniques: field emission scanning electron microscopy (FE-SEM, S-4200, Hitachi, Munich, Germany) coupled with energy dispersive X-ray spectroscopy (EDS) on-system, operating at a voltage of 15 kV. The size distributions of the three samples were identified using a JEOL JEM 2100 high-resolution transmission electron microscope (HRTEM, Pleasanton, CA, USA) operating at 200 kV. Their structural patterns were established using X-ray diffraction (XRD, Cambridge, UK) analysis with Cu Ka radiation at 40 mA and 40 kV, respectively. ThermoGravimetric Analyser 4000 (TGA Perkin Elmer, Johannesburg, South Africa) was performed at temperatures ranging from 30 to 600 °C at a rate of 10 °C min^−1^. Fourier transform infrared spectroscopy (FTIR) analysis was achieved with the aid of a Bruker Platinum ATR Model Alpha (Waltham, MA, USA). PerkinElmer LAMBDA 365 and LS 45 fluorimeters (Waltham, MA, USA) were used to understand the optical properties (UV-Vis and PL analysis) of the three samples. NMR analysis was carried out using a Bruker AV-400 spectrometer (Waltham, MA, USA), working at 400.13 MHz and 300 K, with a spinning rate of 4 kHz.

## 4. Conclusions

In conclusion, a heat-up approach with the aid of coordinating solvent was used to form γ-MnS hexagonal wurtzite structure quantum dots with varying morphologies. FTIR reveals vibration peaks related to MnS vibration modes in all the samples. SEM and HRTEM images reveal a spongy and irregular spherical microstructure with numerous small particles. UV-Vis and photoluminescence show a band-gap energy of 3.78–4.0 eV and emission peaks in the range of 451–602 nm. The purity of the three MnS nanoparticles confirms the presence of Mn and S elements. The well-grown and uniform nanocrystal with an inner hollow structure, smooth outer and inner surfaces and a relatively dense surface indicates that the formed γ-MnS can be explored for energy and storage-device applications.

## Figures and Tables

**Table 1:** Application of MnS nanoparticles prepared from different metal source. PS = present study.

Appearance	Source	Phase	Time	T. °C	Size (nm)	Applications or Potential	Year	Reference
Spherical	MnCl_2_·2H_2_O	MnS_2	30 min		30–40	Photocatalytic hydrogen	2022	[37]
Spherical	MnCl_2_·2H_2_O	MnS	30 min	400	9.19–14.85	Antibacterial activity	2021	[38]
Flower-like	Mn(CH_3_COO)_24_·H_2_O	γ-MnS Mn_3_O_4_	2 h	220	400–600	Photocatalytic activity	2018	[34]
Nanoparticles	Mn(CH_3_COO)_2_·4H_2_O	MnS	3 h	800		Sodium-ion capacitors	2020	[36]
Heterostructures	Mn(CH_3_COO)_2_·4H_2_O	γ-MnS α-MnS MnS	2 h	300	15–20	Optoelectronics	2020	[29]
Nanoparticles	Mn(NO_3_)_2_·4H_2_O	γ-MnS	24 h	90	15	Supercapacitors	2018	[25]
Nanopores	Mn(NO_3_)_2_·4H_2_O	MnS	24 h	25		Optoelectronics	2017	[32]
Spherical	C_4_H_6_MnO_4_·4H_2_O	γ-MnS	2 h	40	21–45	Photoconductors	2017	[26]
Nanoparticles	Mn(NO_3_)_2_	γ-MnS	3 h	85	20–30	Supercapacitors	2017	[35]
Spherical	MnCl₂·4H₂O	γ-MnS	2 h	260	2–15	QDSC	2022	PS

## Data Availability

Not applicable.

## Sample Availability

Samples of the compounds are available from the authors upon request.

## References

[1] Agoro M.A., Meyer E.L., Mbese J.Z., Fuku X., Ahia C.C. (2022). Aliphatic mixed ligands Sn(II) complexes as photon absorbers in quantum dots sensitized solar cell. J. Solid State Chem..

[2] Agoro M.A., Mbese J.Z., Meyer E.L., Onyenankeya K. (2021). Electrochemical signature of CuS photosensitizers thermalized from alkyldithiocarbamato Cu(II) molecular precursors for quantum dots sensitized solar cells. Mater. Lett..

[3] Agoro M.A., Mbese J.Z., Meyer E.L. (2021). Inorganic Pb(II)–P and Pb(II)–S Complexes as Photosensitizers from Primary and Secondary Amines in Dyes-Sensitized Solar Cells. ACS Omega.

[4] Punnoose D., Rao S.S., Kim S.K., Kim H.J. (2015). Exploring the effect of manganese in lead sulfide quantum dot sensitized solar cell to enhance the photovoltaic performance. RSC Adv..

[5] Kumar S., Riyajuddin S., Afshan M., Aziz S.T., Maruyama T., Ghosh K. (2021). In-Situ Growth of Urchin Manganese Sulfide Anchored Three-Dimensional Graphene (γ-MnS@ 3DG) on Carbon Cloth as a Flexible Asymmetric Supercapacitor. J. Phys. Chem. Lett..

[6] Zhang L., Zhou L., Wu H.B., Xu R., Lou X.W. (2012). Unusual Formation of Single-Crystal Manganese Sulfide Microboxes Co-mediated by the Cubic Crystal Structure and Shape. Angew. Chem. Int. Ed..

[7] Liu S., Sankar K.V., Kundu A., Ma M., Kwon J.Y., Jun S.C. (2017). Honeycomb-like interconnected network of nickel phosphide heteronanoparticles with superior electrochemical performance for supercapacitors. ACS Appl. Mater. Interfaces.

[8] Hannachi A., Maghraoui-Meherzi H. (2017). Growth of different phases and morphological features of MnS thin films by chemical bath deposition: Effect of deposition parameters and annealing. J. Solid State Chem..

[9] Gui Y., Qian L., Qian X. (2011). Hydrothermal synthesis of uniform rock salt (α-) MnS transformation from wurtzite (γ-) MnS. Mater. Chem. Phys..

[10] Theerthagiri J., Karuppasamy K., Durai G., Rana A.U.H.S., Arunachalam P., Sangeetha K., Kuppusami P., Kim H.S. (2018). Recent advances in metal chalcogenides (MX; X = S, Se) nanostructures for electrochemical supercapacitor applications: A brief review. Nanomaterials.

[11] Barik R., Ingole P.P. (2020). Challenges and prospects of metal sulfide materials for supercapacitors. Curr Opin Electrochem..

[12] Liu W., Niu H., Yang J., Cheng K., Ye K., Zhu K., Wang G., Cao D., Yan J. (2018). Ternary transition metal sulfides embedded in graphene nanosheets as both the anode and cathode for high-performance asymmetric supercapacitors. Chem. Mater..

[13] Ulutas C., Guneri E., Kirmizigul F., Altindemir G., Gode F., Gumus C. (2013). γ-MnS thin films prepared by chemical bath deposition: Effect of bath temperature on their physical properties. Mater. Chem. Phys..

[14] Gümüş C., Ulutaş C., Ufuktepe Y.Ü.K.S.E.L. (2007). Optical and structural properties of manganese sulfide thin films. Opt. Mater..

[15] Yu X., Li-yun C., Jian-feng H., Jia L., Jie F., Chun-yan Y. (2013). Influence of S/Mn molar ratio on the morphology and optical property of γ-MnS thin films prepared by microwave hydrothermal. J. Alloys Compd..

[16] Zhang Y., Wang H., Wang B., Yan H., Yoshimura M. (2002). Low-temperature hydrothermal synthesis of pure metastable γ-manganese sulfide (MnS) crystallites. J. Cryst. Growth.

[17] Clifford J.N., Planells M., Palomares E. (2012). Advances in high efficiency dye sensitized solar cells based on Ru(II) free sensitizers and a liquid redox electrolyte. J. Mater. Chem..

[18] Bozic-Weber B., Constable E.C., Housecroft C.E. (2013). Light harvesting with Earth abundant d-block metals: Development of sensitizers in dye-sensitized solar cells (DSCs). Coord. Chem. Rev..

[19] Agoro M.A., Meyer E.L., Mbese J.Z., Manu K. (2020). Electrochemical fingerprint of CuS-hexagonal chemistry from (*bis*(*N*-1,4-Phenyl-*N*-(4-morpholinedithiocarbamato)copper(II) complexes) as photon absorber in quantum-dot/dye-sensitised solar cells. Catalysts.

[20] Agoro M.A., Mbese J.Z., Meyer E.L. (2020). Electrochemistry of Inorganic OCT-PbS/HDA and OCT-PbS Photosensitizers Thermalized from *bis*(*N*-diisopropyl-*N*-octyldithiocarbamato) Pb(II) Molecular Precursors. Molecules.

[21] Meyer E.L., Mbese J.Z., Agoro M.A., Taziwa R. (2020). Optical and structural-chemistry of SnS nanocrystals prepared by thermal decomposition of *bis*(*N*-di-isopropyl-*N*-octyldithiocarbamato) tin(II) complex for promising materials in solar cell applications. Opt. Quantum Electron..

[22] Ashbrook L.N., Elliott C.M. (2013). Dye-sensitized solar cell studies of a donor-appended *bis*(2,9-dimethyl-1,10-phenanthroline) Cu(I) dye paired with a cobalt-based mediator. J. Phys. Chem. C.

[23] Yuan Y.J., Yu Z.T., Zhang J.Y., Zou Z.G. (2012). A copper(I) dye-sensitised TiO_2_-based system for efficient light harvesting and photoconversion of CO_2_ into hydrocarbon fuel. Dalton Trans..

[24] Saha R., Sahana A., Lohar S., Banerjee A., Das S., Das D. (2014). pH-controlled solid-phase enrichment of Mn(II): Confirmation of the structure of the extracted ternary Mn(II) complex by single crystal X-ray structure analysis. Desalin. Water Treat..

[25] Arul N.S., Han J.I., Mangalaraj D. (2018). Fabrication of highly flexible conducting electrode based on MnS nanoparticles/graphite/scotch tape for supercapacitor applications. J. Mater. Sci. Mater. Electron..

[26] Chaki S.H., Chauhan S.M., Tailor J.P., Deshpande M.P. (2017). Synthesis of manganese sulfide (MnS) thin films by chemical bath deposition and their characterization. J. Mater. Res. Technol..

[27] Ferretti A.M., Mondini S., Ponti A. (2017). Manganese Sulfide (MnS) Nanocrystals: Synthesis, Properties, and Applications. Advances in Colloid Science.

[28] Dey S., Kumar V.P. (2020). The performance of highly active manganese oxide catalysts for ambient conditions carbon monoxide oxidation. Curr. Opin. Green Sustain. Chem..

[29] Kadhm A.J., Atwan A.F., Ismail R.A. (2021). Effect of molar concentration on the structural, optical and electrical properties of the MnS thin film prepared by spray pyrolysis. J. Phys. Conf. Ser..

[30] Heiba Z.K., Mohamed M.B., Ahmed S.I., El-Naggar A.M., Albassam A.A. (2020). Effect of composition ratio on the structural and optical properties of MnS@ ZnS nanocomposites. J. Mater. Sci. Mater. Electron..

[31] Elango M., Gopalakrishnan K., Vairam S., Thamilselvan M. (2012). Structural, optical and magnetic studies on non-aqueous synthesized CdS: Mn nanomaterials. J. Alloys Compd..

[32] Hu D., Zhang Y., Lin J., Hou Y., Li D., Wu T. (2017). Dual emissions from MnS clusters confined in the sodalite nanocage of a chalcogenide-based semiconductor zeolite. Dalton Trans..

[33] Chen Z.T., Song E.H., Wu M., Zhou B., Zhang Q.Y. (2016). Exchange coupled Mn-Mn pair: An approach for super-broadband 1380 nm emission in α-MnS. Appl. Phys. Lett..

[34] Zhang J., Shi R., Zhang C., Li L., Mei J., Liu S. (2018). Solvothermal synthesis of manganese sulfides and control of their phase and morphology. J. Mater. Res..

[35] Kumbhar V.S., Lee Y.R., Ra C.S., Tuma D., Min B.K., Shim J.J. (2017). Modified chemical synthesis of MnS nanoclusters on nickel foam for high performance all-solid-state asymmetric supercapacitors. RSC adv..

[36] Li S., Chen J., Xiong J., Gong X., Ciou J., Lee P.S. (2020). Encapsulation of MnS nanocrystals into N, S-Co-doped carbon as anode material for full cell sodium-ion capacitors. Nanomicro Lett..

[37] Priya B.A., Sivakumar T., Venkateswari P. (2022). Controlled loading of MnS_2 on porous TiO_2_ nanosheets for enhanced photocatalytic hydrogen evolution. J. Mater. Sci. Mater. Electron..

[38] Jeyamalar K., Selvarajan P. (2021). Studies of magnesium doped MnS nanomaterial synthesized by microwave-assisted solution method. J. Adv. Sci. Res..

[39] Balpinar N., Göde F. (2020). Fabrication and characterization of dye-sensitized solar cells based on murexid dye and inorganic cds: Mn Thin Films. Chalcogenide Lett.

[40] Akman E. (2020). Enhanced photovoltaic performance and stability of dye-sensitized solar cells by utilizing manganese-doped ZnO photoanode with europium compact layer. J. Mol. Liq..

[41] Alavi M., Rahimi R., Maleki Z., Hosseini-Kharat M. (2020). Improvement of power conversion efficiency of quantum dot-sensitized solar cells by doping of manganese into a ZnS passivation layer and cosensitization of zinc-porphyrin on a modified graphene oxide/nitrogen-doped TiO_2_ photoanode. ACS Omega.

[42] Fadaam S.A., Ali H.M., Shaban A.H., Ahmed S.A. (2020). December. Improving efficiency of solar cell for MnS through annealing. AIP Conf. Proc..

[43] Cao X., Ding R., Zhang Y., Cui Y., Hong K. (2021). A heterojunction film of NiS_2_ and MnS as an efficient counter electrode for dye-sensitized solar cells. Mater. Today Commun..

[44] Beltran-Huarac J., Palomino J., Resto O., Wang J., Jadwisienczak W.M., Weiner B.R., Morell G. (2014). Highly-crystalline γ-MnS nanosaws. RSC Adv..

[45] Zhang N., Yi R., Wang Z., Shi R., Wang H., Qiu G., Liu X. (2008). Hydrothermal synthesis and electrochemical properties of alpha-manganese sulfide submicrocrystals as an attractive electrode material for lithium-ion batteries. Mater. Chem. Phys..

